# Guano exposed: Impact of aerobic conditions on bat fecal microbiota

**DOI:** 10.1002/ece3.4084

**Published:** 2018-04-27

**Authors:** Viacheslav Y. Fofanov, Tara N. Furstenau, Daniel Sanchez, Crystal M. Hepp, Jill Cocking, Colin Sobek, Nicole Pagel, Faith Walker, Carol L. Chambers

**Affiliations:** ^1^ School of Informatics, Computing, and Cyber Systems Northern Arizona University Flagstaff Arizona; ^2^ Pathogen and Microbiome Institute Northern Arizona University Flagstaff Arizona; ^3^ School of Forestry Northern Arizona University Flagstaff Arizona

**Keywords:** abiotic effects, bats, guano microbiota, noninvasive sampling, postdefecation

## Abstract

Bats and their associated guano microbiota provide important terrestrial and subterranean ecosystem services and serve as a reservoir for a wide range of epizootic and zoonotic diseases. Unfortunately, large‐scale studies of bats and their guano microbiotas are limited by the time and cost of sample collection, which requires specially trained individuals to work at night to capture bats when they are most active. Indirectly surveying bat gut microbiota through guano deposits could be a more cost‐effective alternative, but it must first be established whether the postdefecation exposure to an aerobic environment has a large impact on the guano microbial community. A number of recent studies on mammalian feces have shown that the impact of aerobic exposure is highly species specific; therefore, it is difficult to predict how exposure will affect the bat guano microbiota without empirical data. In our study, we collected fresh guano samples from 24 individuals of 10 bat species that are common throughout the arid environments of the American southwest and subjected the samples to 0, 1, and 12 hr of exposure. The biodiversity decreased rapidly after the shift from an anaerobic to an aerobic environment—much faster than previously reported in mammalian species. However, the relative composition of the core guano microbiota remained stable and, using highly sensitive targeted PCR methods, we found that pathogens present in the original, non‐exposed samples could still be recovered after 12 hr of exposure. These results suggest that with careful sample analysis protocols, a more efficient passive collection strategy is feasible; for example, guano could be collected on tarps placed near the roost entrance. Such passive collection methods would greatly reduce the cost of sample collection by allowing more sites or roosts to be surveyed with a fraction of trained personnel, time, and effort investments needed.

## 1 INTRODUCTION

Chiroptera is a large and widespread mammalian order with more than 1,300 species of bats distributed worldwide, absent only in the Arctic, Antarctic, and some island chains. Bats provide important ecosystem services as pollinators, seed dispersers, and pest control agents (Kunz, de Torrez, Bauer, Lobova, & Fleming, 2011), and they serve as the foundation species in subterranean habitats (caves and abandoned mines) where their regular deposition of nutrient‐rich guano supports a diverse food web (Emerson & Roarki, 2007; Ferreira & Martins, 1998; Salgado, Motta, De Souza Aguiar, & Nardoto, 2014). Bats are also increasingly recognized as an important reservoir for a number of human‐ and livestock‐affecting viral (Krebs, Mandel, Swerdlow, & Rupprecht, 2004), bacterial (Brouqui & Raoult, 2006; Mogollon‐Pasapera, Otvos, Giordano, & Cassone, 2009; Veikkolainen, Vesterinen, Lilley, & Pulliainen, 2014), and fungal pathogens (Daszak, 2000; Wood et al., 2012). Many of these pathogens have been detected in bat guano, which is a likely source of zoonosis (Muhldorfer, 2013). Because bats are important contributors to ecological processes and the spread of disease, it is important to monitor and understand bats and their associated microbiota, which can provide valuable insights into their life history, physiology, food sources, geographical movements, and disease state.

Recent advances in high‐throughput sequencing and PCR techniques targeting the 16S rRNA gene (Weisburg, Barns, Pelletier, & Lane, 1991) for bacteria, ITS (Seifert, 2009) for fungi, COI (Hebert, Cywinska, Ball, & Dewaard, 2003) and custom DNA barcodes (Ratnasingham & Hebert, 2007) for eukaryotes have greatly reduced the time and labor involved in studying microbiotas and their effect on biological processes in wildlife. With these techniques, questions about diet (Bohmann et al., 2011; Razgour et al., 2011; Zeale, Butlin, Barker, Lees, & Jones, 2011), microbiota (Banskar, Bhute, Suryavanshi, Punekar, & Shouche, 2016), and diseases (both epizootic and zoonotic (Taylor, Latham, & Woolhouse, 2001; Woolhouse & Gowtage‐Sequeria, 2005)) can be answered relatively quickly and efficiently. While significant technological progress has been made in processing and analyzing samples collected from wildlife, obtaining consistent, reliable, and unbiased samples remains a costly and time‐consuming challenge. This is especially true for free‐ranging wildlife species that occur in remote and difficult to access habitats. Actively collecting samples from such species requires trained personnel, working long hours (often at night), tracking, and trapping the animals. This process is slow, unreliable, provides limited sample sizes (for some species), and is expensive. As a result, sample collection represents a significant bottleneck in systematic and consistent wildlife surveillance and research programs (Grogan et al., 2014; Stallknecht, 2007).

Some of the challenges of active collection can be avoided through indirect sampling methods. For many wild and elusive species, feces‐based surveillance is commonly applied because it does not require animal disturbance, capture, or euthanasia (Walker, Horsup, & Taylor, 2009; Walker, Williamson, Sanchez, Sobek, & Chambers, 2016). For many communal species with stable home ranges or roosting behaviors (including some bat species), feces are typically abundant, easy to spot, and, depending on location (not deep inside abandoned mines or caves), require no significant training to collect. Such collection techniques have already been employed for monitoring endangered (e.g., gorillas (*Gorilla gorilla*) (Etienne et al., 2012; Liu et al., 2010)) or elusive wildlife (e.g., chimpanzees (*Pan troglodytes*) (Harvala et al., 2011; Makuwa et al., 2005) and bats (Walker et al., 2016)), largely alleviating technical and logistic challenges in obtaining samples from free‐ranging populations (Stallknecht, 2007).

In many wildlife applications, feces are not collected immediately after defecation and often hours or days may pass with feces exposed to environmental conditions prior to sample stabilization. Thus, the biggest weakness of indirect, feces‐based wildlife monitoring approaches for studying microbiota is the degradation of the fecal communities due to the shift from anaerobic (gut) to aerobic (outside gut) environments. A number of studies have attempted to quantify the change in mammalian fecal microbiota due to sample exposure in Giraffes (*Giraffa camelopardalis*) (Menke, Meier, & Sommer, 2015), Springboks (*Antidorcas marsupialis*) (Menke et al., 2015), humans (*Homo sapiens*) (Dominianni, Wu, Hayes, & Ahn, 2014; Flores et al., 2015), and cows (*Bos taurus*) (Vo & Jedlicka, 2014; Wong et al., 2016). The results from each of these studies varied, with feces from some species exhibiting drastic microbiota changes within days of defecation (Menke et al., 2015; Wong et al., 2016) and others remaining relatively stable for weeks (Dominianni et al., 2014; Flores et al., 2015; Menke et al., 2015). Combined, these studies suggest that the stability of fecal microbiota is highly dependent on the wildlife species and, likely, permeability of feces to aerobic environment. This means that suitability of fecal material for indirect, noninvasive monitoring approaches will likely need to be established or verified for each species or genus before accurate interpretations of fecal microbiota can be made.

Large deposits of bat guano often arise inside or beneath roosts and in surrounding flight corridors. These deposits provide easy access to guano that do not require invasive procedures such as capturing bats. If guano samples from such deposits are useful for feces‐based surveillance, it could significantly ease logistics of sample collection and enable cost‐effective, large‐scale studies of bat communities. Such studies could offer insights into the state of gut microbiotas, pathogen transmission for pathogens shed in feces, and diet, provided that the stability of the fecal microbiota is not significantly impacted by environmental exposure. Therefore, our goal in this study was to establish whether samples from guano deposits are an appropriate alternative to freshly collected samples by analyzing changes in composition and biodiversity of bat guano due to short‐ and medium‐length exposure.

## 2 MATERIALS AND METHODS

### 2.1 Sample acquisition and processing

We collected fresh guano samples from bats in July 2015 at the Canyon de Chelly National Monument. Bats were captured via traditional mist netting methods (Kunz & Kurta, 1988). We captured and handled animals under guidelines of the American Society of Mammalogists (Sikes & Mammal, 2016) and with approval of Northern Arizona University (NAU) Institutional Animal Care and Use Committee (permit number 07‐006‐R2), Arizona Game and Fish Department (permit SP706855), and the Navajo Nation (Special Permit 908). Trapping started at dusk and continued until captures decreased to <1 bat per 30 min. Captured bats were held for 10 min or less and were released after collection of genetic samples. No bats suffered injury or mortality in this study. We identified species via visual inspection and later confirmed identification using our barcoding techniques (Walker et al., 2016). Captured bats were placed in a fresh, bleach‐sterilized cotton cloth bag for up to 5 min, and guano pellets were then collected directly from the bat or from the bag. Each guano pellet (≥1 pellet(s) per bat) was separated in the field into three equal portions. In cases where bats produced fewer than three pellets (e.g., EUMA03 sample), the pellet was divided using a sterile inoculating loop to slice the pellet. One portion was immediately stabilized in RNAlater within a 5‐ml conical tube. The remaining two portions were left exposed to the elements (19°C average temperature, 51% humidity, 1,709.9 m elevation above sea level) in an open conical tube for 1 or 12 hr. Once the desired exposure time was reached, samples were stabilized in RNAlater. DNA was extracted from all samples using the QIAamp Fast DNA Stool Kit, and all extractions were stored at −80°C upon arrival in the laboratory, within 48 hr of collection.

### 2.2 16S rRNA amplicon sequencing and analysis

Taxonomic groups within the Bacteria and Archaea domains were identified using amplicon sequencing targeting the 16S rRNA gene (Weisburg et al., 1991). The standard 341F/785R primers (Klindworth et al., 2013) were used for the broadest coverage of guano microbiota, and amplicons were sequenced using 2 × 300 base reads on an Illumina MiSeq (Weisburg et al., 1991). Two runs were used to generate sequence data, and no statistically significant run effects were detected. Quantitative Insights into Microbial Ecology (QIIME) software package (Caporaso et al., 2010b) was used with default parameters (see Appendix S1 for list of parameters) to analyze the microbiota data and statistically test the impact of our explanatory variables on the metadata. Briefly, open‐reference OTU picking was performed using the UCLUST algorithm (Edgar, 2010) and reads were aligned to the Greengenes reference set (Desantis et al., 2006) version 13_8 with 97% OTU representative sequence clusters using PyNAST (Caporaso et al., 2010a). For the core diversity analyses, rarefaction was performed using a minimum read depth of 4,800. FastTree (Price, Dehal, & Arkin, 2009) was used for phylogenetic tree reconstruction to provide insight into the evolutionary relationships between the OTUs and for use in phylogenetic diversity calculations. Weighted and unweighted UniFrac distance metrics (Lozupone, Hamady, Kelley, & Knight, 2007; Lozupone & Knight, 2005) were used to quantify changes in the guano microbiota upon exposure to the elements; these two metrics target changes in relative frequencies and absolute presence/absence of bacteria, respectively. We used Web‐PHYLIP (Lim & Zhang, 1999) to generate neighbor‐joining trees from the UniFrac distance matrices. SourceTracker (Henry et al., 2016) was used to quantify the degree to which microbiota from the original source sample (time 0) could explain the exposed sample's bacterial composition. SourceTracker was run using default parameters after filtering OTUs that are present in less than two samples. Three different source/sink relationships were assessed for each sample: hour 0 versus hour 1, hour 0 versus hour 12, and hour 1 versus hour 12. We measured alpha diversity using the Shannon index, and diversity differences between the different exposure times were analyzed using a two‐sided paired *t*‐test from the R stats package (R Development Core Team, 2013) with the significance level at 0.05.

### 2.3 Targeted TaqMan assays and analysis

The extracted DNA from the original and exposed samples was analyzed using TaqMan assays (Holland, Abramson, Watson, & Gelfand, 1991) to assess the presence and relative amounts of two facultative anaerobic bacterial pathogens: *Escherichia coli* and *Pseudomonas aeruginosa*. These bacteria are routinely found in our fresh guano samples and are useful sentinel targets for assessing the impact of exposure on pathogen detection, at least for facultative anaerobes. For detection of *E. coli*, the following reaction mix was combined for a final volume of 10 μl: 1 μl of the extracted DNA, 1.77 μl of sterile, molecular grade water, 5 μl of 2× TaqMan Universal PCR Master Mix (Life Technologies, CA, USA; p/n 4440040), 0.3 μl of each 20 μmol/L primer, and 0.13 μl of the 20 μmol/L probe. For detection of *P. aeruginosa*, the following reaction mix was combined for a final volume of 10 μl: 1 μl of the extracted DNA, 2.55 μl of sterile, molecular grade water, 5 μl of 2× TaqMan Universal PCR Master Mix (Life Technologies; p/n 4440040), 0.6 μl of each 10 μmol/L primer, and 0.25 μl of the 10 μmol/L probe. In all cases, real‐time PCR conditions were as follows: 50°C for 2 min, 95°C for 10 min, then 40 cycles of 95°C for 15 s, and 60°C for 1 min. The reaction was run using the Applied Biosystems 7900HT Fast real‐time PCR system. The results were evaluated using SDS v2.4 software, which automatically set the baseline and the threshold to 0.17.

## 3 RESULTS

**Figure 1:**
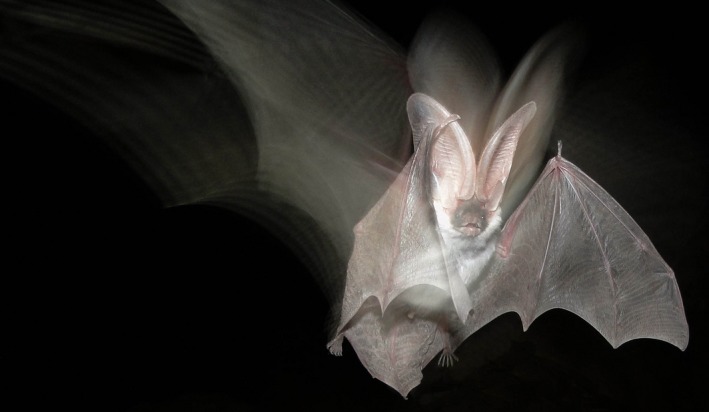
Flying male spotted bat (*Euderma maculatum*)

We obtained fresh guano samples from 24 bats. These individuals represented 10 of 28 species found in Arizona (Table 1). Captured bats included Mexican free‐tailed bat (*Tadarida brasiliensis*) and big brown bat (*Eptesicus fuscus*), two of the most abundant bat species on Arizona's Colorado Plateau, as well as spotted bat (*Euderma maculatum*, Figure 1) and big free‐tailed bat (*Nyctinomops macrotis*)—both rarely captured species. Combined, these species represent a substantial portion of bat biodiversity on the Colorado Plateau, with a wide range of diets.

**Table 1:** Bat species guano samples collected during the sampling event

Abbreviation	Scientific name	Common name	No. of samples
ANPA	*Antrozous pallidus*	Pallid bat	1
EPFU	*Eptesicus fuscus*	Big brown bat	1
EUMA	*Euderma maculatum*	Spotted bat	1
MYCA	*Myotis californicus*	California myotis	4
MYEV	*Myotis evotis*	Long‐eared myotis	1
MYVO	*Myotis volans*	Long‐legged myotis	3
MYYU	*Myotis yumanensis*	Yuma myotis	3
NYMA	*Nyctinomops macrotis*	Big free‐tailed bat	1
PAHE	*Parastrellus hesperus*	Canyon bat	1
TABR	*Tadarida brasiliensis*	Mexican free‐tailed bat	8

### 3.1 Impact of exposure on guano microbiota diversity

**Figure 2:**
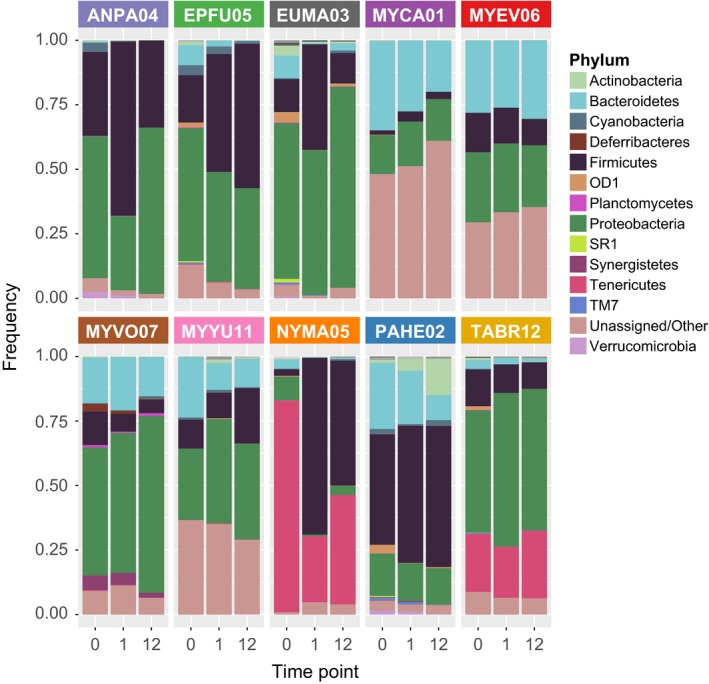
Phylum‐level taxonomic summary for guano samples from 10 bat species with 0, 1, or 12 hr of exposure. The height of the stacked bars represents the proportion of each taxon in the sample. Low‐frequency taxa (less than 0.01 across all samples) are shown in gray scale at the top of the bar and are not labeled by name

**Figure 3:**
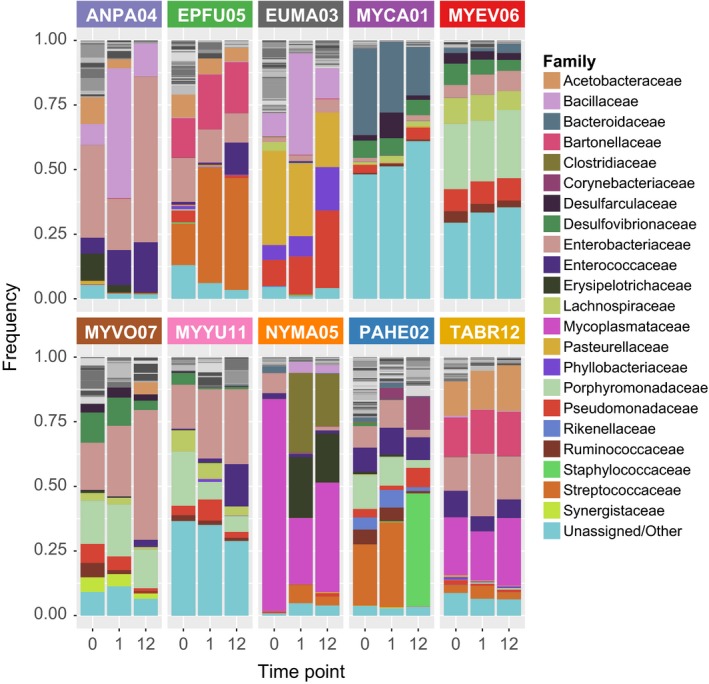
Family‐level taxonomic summary for guano samples from 10 bat species with 0, 1, and 12 hr of exposure. The height of the stacked bars represents the proportion of each taxon in the sample. Low‐frequency taxa (less than 0.05 across all samples) are shown in gray scale at the top of the bar and are not labeled by name

The guano microbiota profiles varied across the 10 species that were analyzed in this study. At the phylum level, Proteobacteria (ANPA04: 55%, EPFU05: 52%, EUMA03: 60%, MYCA01: 15%, MYEV06: 27%, MYVO07: 50%, MYYU11: 28%, NYMA05: 9%, PAHE02: 16%, and TABR12: 47%), Firmicutes (ANPA04: 32%, EPFU05: 18%, EUMA03: 13%, MYCA01: 2%, MYEV06: 15%, MYVO07: 13%, MYYU11: 11%, NYMA05: 2%, PAHE02: 43%, and TABR12: 14%), and Bacteroidetes (ANPA04: 0.05%, EPFU05: 8%, EUMA03: 9%, MYCA01: 35%, MYEV06: 28%, MYVO07: 18%, MYYU11: 23%, NYMA05: 3%, PAHE02: 26%, and TABR12: 3%) were abundant in most of the samples before exposure (Figure 2). MYCA01, MYEV06, and MYYU11 had high proportions of unassigned sequences and Tenericutes were abundant only in NYMA05 (82%) and TABR12 (22%), both of which are in the Free‐tailed (Molossidae) family. In most cases, there were minor variations in the abundance of major phyla with 1 and 12 hr of exposure; the exceptions were the NYMA05 sample, which showed a decrease in Tenericutes and an increase in Firmicutes upon exposure, and the ANPA04 sample, which showed a decrease in Proteobacteria and an increase in Firmicutes at 1‐hr exposure that reversed at 12 hr of exposure. The microbiota profiles at the family level (Figure 3) show greater variation in the composition across the different species, and the pre‐exposure samples generally have a larger proportion of low‐frequency taxa (gray scale) compared to the exposed samples.

**Figure 4:**
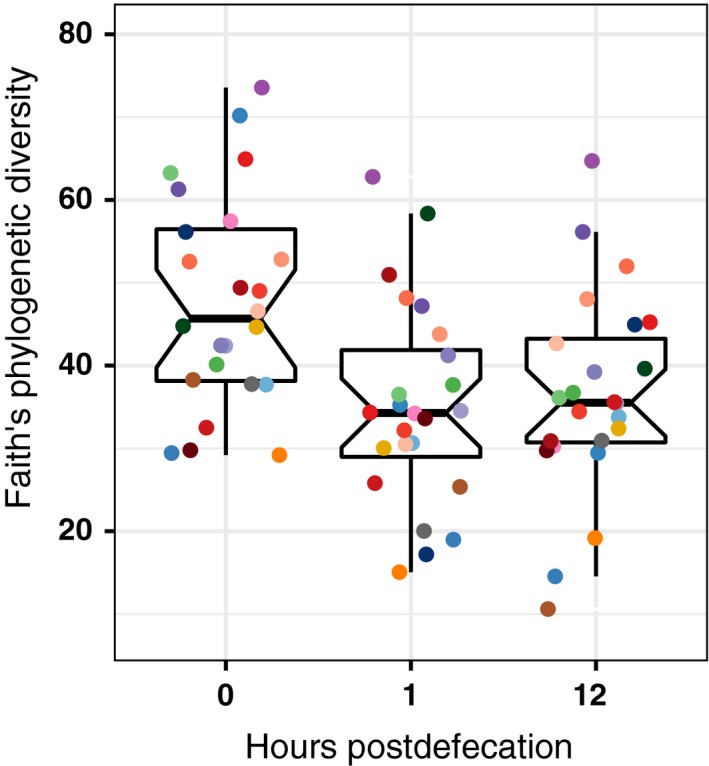
Phylogenetic diversity of bat guano microbiota decreases substantially as soon as 1 hr postdefecation, and although diversity rebounds slightly after 12 hr postdefecation, the overall trend is still downward. The points represent Faith's phylogenetic diversity measures for samples from all species (*n *=* *24) at 0, 1, and 12 hr postdefecation. The upper and lower hinges of the box‐and‐whisker plots represent the first and third quartiles, and the whiskers extend beyond the hinges by 1.5× IQR. The notches extend to 1.58 × IQR/√*n* providing roughly a 95% confidence interval for the median. Each color indicates a different sample matching the color scheme in Figure 6

We observed a substantial drop in guano microbiota diversity even with brief exposure to ambient conditions. We measured Faith's phylogenetic diversity (Faith, 1992) in guano microbiota from each of the 24 samples at 0 (*M *=* *5.04, *SD* = 1.25), 1 (*M *=* *4.60, *SD* = 1.01), and 12 (*M *=* *4.41, *SD* = 0.76) hours postdefecation and observed a significant decrease, *t*(23) = 2.41, *p* = .02, $\overset{¯}{d}$ = 0.43, 95% CI 0.06, 0.81, Cohen's *d *=* *0.49, in the diversity between 0 and 1 hr (Figure 4). There was a nonsignificant increase in diversity between hour 1 and hour 12, *t*(23) = 1.62, *p *=* *.12, $\overset{¯}{d}$ = 0.19, 95% CI −0.05 to 0.44, Cohen's *d *=* *0.33, largely due to an increase in aerobic bacteria. The overall trend between hour 0 and hour 12 showed a significant decrease in diversity, *t*(23) = 3.44, *p *=* *.002, $\overset{¯}{d}$ = 0.63, 95% CI 0.25–1.00, Cohen's *d *=* *0.70, with exposure time which occurred primarily within the first hour. The initial decrease in biodiversity was likely due to the rapid environmental transition upon defecation, particularly the shift from an anoxic to oxic environment. To corroborate this, we compared ratios of the frequency of anaerobes to the frequency of aerobes and facultative anaerobes between the 0‐ and 1‐hr samples. We found that the ratio decreased from 0.81 (interquartile range (IQR) = 1.43) at time 0 to 0.58 (IQR = 0.48) at 1 hr, which suggests that the initial shift into an oxygen‐rich environment reduced biodiversity among anaerobes.

**Figure 5:**
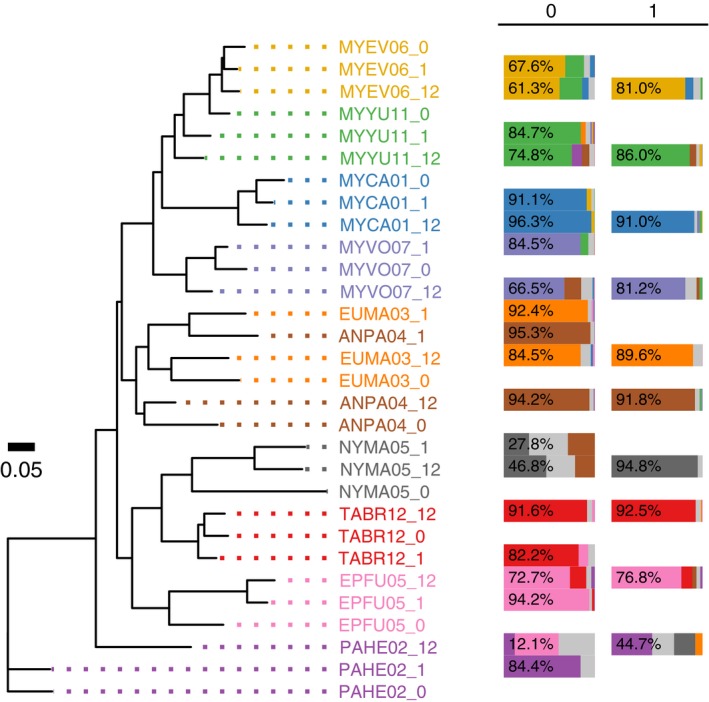
Ambient‐exposed and immediately stabilized samples from the same individual consistently group together (7/10) and a large proportion of the guano microbiota composition of the ambient‐exposed samples are explained by the original source sample. The neighbor‐joining tree (left) is based on weighted UniFrac distances measured for guano samples from 10 bat species collected at 0, 1, and 12 hr postdefecation (samples indicated in the taxon name by: _0, _1, or _12). SourceTracker results (right) show the degree to which the guano microbiota from exposed samples is explained by the source bacterial composition. The colors in the bar charts represent the proportion of the guano microbiota composition that is explained by each of the source samples. The source samples in the first column are the time 0 samples and the source samples in the second column are the 1‐hr samples. The proportion of the sample that is explained by the matching source is shown in the leftmost segment of the bar, and the percentage is indicated. The remaining segments of the bar represent sources from other individuals, and the color corresponds to the color of the sample labels in the tree; light gray indicates an unknown source

**Figure 6:**
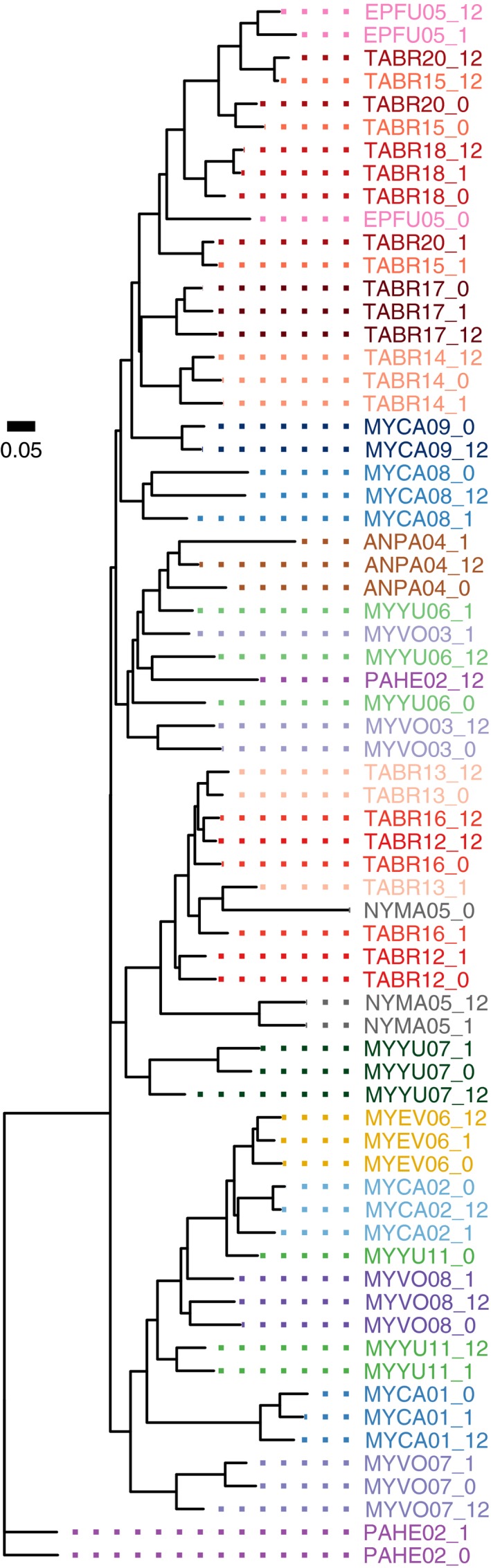
Neighbor‐joining tree based on weighted UniFrac distances measured for 23 guano samples from 10 bat species at 0, 1, and 12 hr postdefecation. In 10 cases, all the sample times form a distinct clade and in an additional four cases, the hour 0 and hour 12 samples form a clade. The MYCA09 (California myotis) 1‐hr sample and all the EUMA (spotted bat) samples were excluded from this analysis because their read counts did not meet the cutoff threshold of 10,150 reads

When the distance between the samples is measured using the relative abundance of OTUs (weighted UniFrac), the guano microbiota similarity between the guano samples collected from the same individual, regardless of exposure time, grouped together and appeared more related to each other than to samples from other individuals. This pattern suggests that the dominant guano microbiota in the sample is preserved; otherwise, it would have been more difficult to identify relationships between exposed and fresh samples from the same individual. Conservation of guano microbiota composition between fresh and exposed samples can be seen from the neighbor‐joining tree generated using weighted UniFrac distances for 10 samples (one from each species; Figure 5 [left]). Similar conservation was observed when all 24 samples were compared to each other (Figure 6). In seven of the 10 individuals, the 0‐, 1‐, and 12‐hr samples formed a clade (Figure 5 [left]). In the remaining individuals, the three samples did not form a distinct clade; however, for EUMA03 (spotted bat) and ANPA04 (pallid bat), the 0‐ and 12‐hr samples do group together suggesting that there were recognizable similarities after 12 hr of exposure.

**Figure 7:**
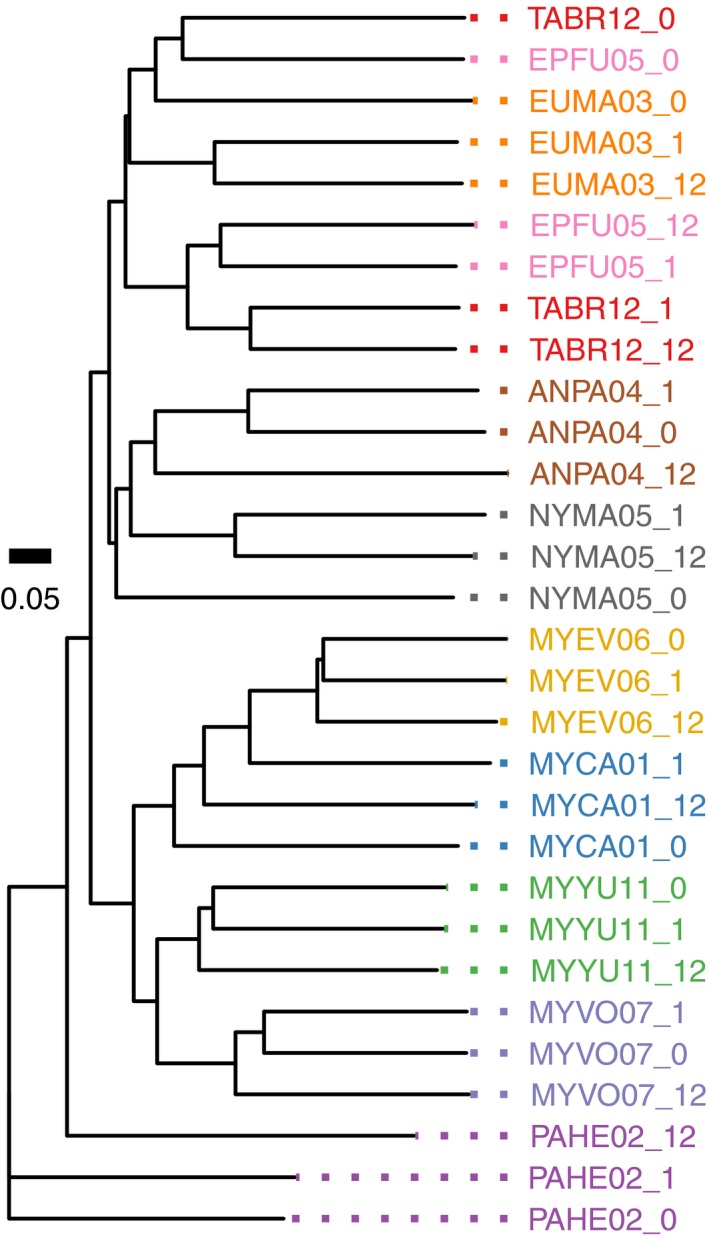
Exposed and unexposed samples from the same individual group together less consistently (5/10) when guano microbiota similarity is based on the presence or absence of OTUs. Neighbor‐joining tree based on unweighted UniFrac distance measured for guano samples from 10 bat species collected at 0, 1, and 12 hr postdefecation

**Figure 8:**
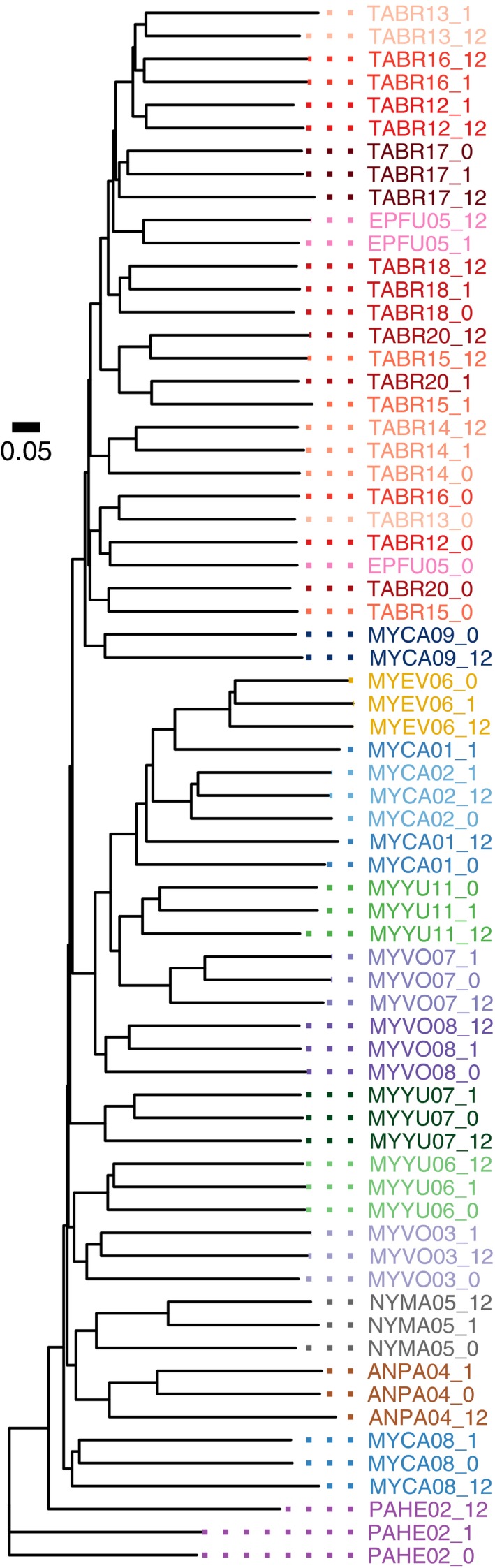
Neighbor‐joining tree based on unweighted UniFrac distances measured for 23 guano samples from 10 bat species at 0, 1, and 12 hr postdefecation. There were 16 cases where all of the sample times form a distinct clade. The MYCA09 (California myotis) 1‐hr sample and all of the EUMA (spotted bat) samples were excluded from this analysis because their read count did not meet the cutoff threshold of 10,150 reads

It was more difficult to correctly group exposed samples with their original sample using the unweighted UniFrac distance measure because there were more substantial changes in the presence and absence of taxa upon exposure (loss of detection of low‐frequency anaerobes). The neighbor‐joining tree generated using unweighted UniFrac distances for the same 10 samples from Figure 5 is shown in Figure 7, and a tree of all the samples is shown in Figure 8. In about half of the cases, the 0‐, 1‐, and 12‐hr samples from the same individual grouped together to form a clade; in the remaining cases (TABR12—Mexican free‐tailed bat, EPFU05—big brown bat, EUMA03—spotted bat, NYMA05—big free‐tailed bat, and MYCA01—California myotis), the 1‐ and 12‐hr exposure samples often formed a clade separate from the unexposed sample. Samples from the same species (viz. the eight TABR individuals) appeared more related using the unweighted UniFrac distance (Figure 8) compared to the weighted UniFrac distance (Figure 6).

Despite absolute and relative frequency changes in guano microbiota composition, in most cases it was still possible to identify the source sample—stabilized at time 0—from which the 1‐ and 12‐hr samples originated. We used SourceTracker to quantify which of the source samples best explained the guano microbiota of each of the exposed samples. In almost every case, the major proportion of the exposed samples was explained by the source sample from the same individual (Figure 5 [right]). The composition of the 12‐hr samples was best explained (by ≥50%) by the corresponding time 0 source with a few exceptions: NYMA05 was at 46.8%, and PAHE02 was low at 12.1%. NYMA05 (big free‐tailed bat) and PAHE02 (canyon bat) also behaved poorly when we analyzed different sources and sinks. The NYMA05 time 0 sample only explained 27.8% of the NYMA05 1‐hr sample, whereas the NYMA05 1‐hr sample explained 94.8% of the NYMA05 12‐hr sample, a discrepancy which suggests that the issue arose in the hour zero sample. Upon closer examination, the time 0 sample contained a much higher percent of Tenericutes (82.1%) and a much lower percent of Firmicutes (2.5%) compared to the 1‐hr (25% and 68.4%, respectively) and 12‐hr (42.4% and 48.4%, respectively) samples. The 1‐hr PAHE02 sample explained a larger percent (44.7%) of the 12‐hr sample than the 0‐hr sample, but it was still less than half. Both of the PAHE02 exposed samples had a large portion of unknown source. The 12‐hr PAHE02 sample contained a much higher percent (14.1%) of Actinobacteria compared to all other samples and the 1‐hr PAHE02 sample contained the second highest percent (4.7%).

### 3.2 Pathogen detection

TaqMan assays were used to determine whether pathogens (*E. coli* and *P. aeruginosa*) detected in the original sample could still be detected after 12 hr of exposure. Using previously validated TaqMan assays, we analyzed the 0‐, 1‐, and 12‐hr samples from 24 individuals and found 10 samples were positive for *E. coli* with an average cycle threshold (Ct) value of 35.13 (*SD* = 1.69) and 43 samples were positive for *P. aeruginosa* with an average Ct value of 32.78 (*SD* = 2.66). We found that 100% of the *E. coli*‐positive samples at time 0 were also positive after 12 hr of exposure (100% specificity) and 100% of samples that were negative at time 0 remained negative after 12 hr (100% sensitivity, Table 2a). False positives and false negatives occurred when detecting *P. aeruginosa* in the samples (Table 2b); specificity was reduced to 75% and sensitivity was 37.5%. The 10 false positives occurred when *P. aeruginosa* was not detected at time 0 but was detected after 12 hr. In each of the false‐positive cases, the sample at 1 hr also tested positive for *P. aeruginosa* so perhaps the pathogen was present at time zero but it was below the level of detection. Two of the samples were false negatives meaning that the samples were positive at time zero but were negative at 12 hr.

**Table 2:** Pathogens detected at time zero were detected after 12‐hr exposure 100% of the time for (a) *E. coli* and 75% of the time for (b) *P. aeruginosa*

	0+	0−
(a) *E. coli*		
12+	4	0
12−	0	20
(b) *P. aeruginosa*		
12+	6	10
12−	2	6

Contingency tables for pathogen detection outcomes for *E. coli* (a) and *P. aeruginosa* (b). The columns of each table represent the pathogen detection status at time zero, and the rows indicate whether the pathogen was detected after 12 hr of exposure. The values in each table sum to a total of 24 samples.

## 4 DISCUSSION

### 4.1 The challenges of active guano microbiota surveillance in bats

Surveillance of wildlife populations has always been difficult (Grogan et al., 2014; Stallknecht, 2007), and this is particularly true for bats because sample collection strategies that are viable in other species are often unsuccessful in bats. For example, passive carcass submission methods (where veterinarians and citizens submit deceased animals for testing) provide an effective and low‐cost solution for many wildlife studies. For bat species however, such surveillance approaches are often unreliable (Mann et al., 2013; Phillips, Stallknecht, Perkins, McClure, & Mead, 2014) because bat and human populations rarely intersect. Many bats dwell in isolated locations (e.g., caves, abandoned mines), and they are mostly nocturnal so they often only hunt and forage at night. Many zoonotically and epizootically relevant pathogens (e.g., *Bartonella*,* Histoplasma*) (Calisher, Childs, Field, Holmes, & Schountz, 2006; Muhldorfer, 2013; Wang et al., 2006) do not cause significant die‐offs so carcasses are not available for collection. Furthermore, the protected status and “uncharismatic” nature of bats may lead to substantial biases, which cause bats to be underrepresented in surveillance efforts (Grogan et al., 2014; Stallknecht, 2007). Pathogen testing and detection frequency have also been observed to strongly correlate with human population size (Childs, Krebs, Real, & Gordon, 2007), and the elusive nature and human‐shy nature of bats only further complicate this problem. Given these factors, it may seem that entering the animal habitat and actively colleting samples are the only viable option.

Active surveillance via bat capture requires special equipment (e.g., mist nets) and is labor‐intensive, requiring the effort of multiple people per site, working late hours. The personnel must be specially trained to handle bats and immunized (e.g., rabies vaccinations) to minimize the risk of disease. Roost site cohabitation of common (e.g., long‐eared myotis) and sensitive species (e.g., Townsend's big‐eared bat) only further complicates permit acquisition and consistency of sample collection efforts out of concern of injuring bats. Similarly, the maternity season (when female bats congregate to give birth and rear their pups) often interrupts sampling to avoid habitat or colony disruption. These challenges, combined with the labor‐intensive and highly variable (0–50 bats per night) nature of active capture approaches, particularly for smaller colony‐forming bats common in the southwestern United States, make it difficult to extend these practices to large‐scale, sustained surveillance efforts.

### 4.2 Guano exposed—implications for indirect surveillance techniques

**Figure 9:**
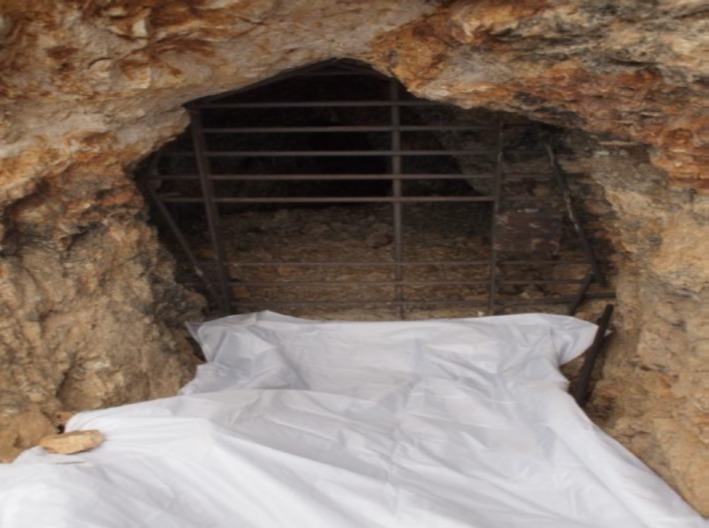
Tarp‐based, indirect fecal sampling of a bat roost located in an abandoned mine in southern Arizona, USA. Tarps can be laid out at the entrance of the roost to catch guano deposited by bats returning from nightly hunting and foraging. Multiple roosts can be sampled each night, by a single researcher. Guano pellets can be collected, catalogued, and stabilized each morning allowing for less than 8–12 hr of exposure, with only moderate disruption to guano microbiota and minimal disruptions in pathogen detection by highly sensitive PCR molecular techniques

Indirect guano‐based sampling techniques can alleviate the scaling issues associated with active surveillance. One such technique is tarp‐based sampling (Figure 9). Tarps, disinfected by a simple bleach solution, can be laid on the ground at the entrance or base of each roost (common roosts in the American Southwest include abandoned mines, caves, snags, cliffs, rocks, and houses) each night. Guano pellets are deposited on the tarps by bats returning from nightly hunting and foraging, and the tarps are collected each morning. Typically, this means that guano will be exposed to the elements for under 8–12 hr. Such approaches do not require specialized training to capture bats or enter mines or caves, do not require additional equipment (night vision equipment, mist nets, caving equipment), can be performed by a single individual, and, provided the roosts are sufficiently close to each other, can be used to sample multiple sites per night. Because minimal disruption to the roost or individual bats is expected from this sample collection methodology, surveillance can occur even at sites suspected to harbor endangered species or sites of maternity roosts.

The effect that the 8–12 hr of exposure has on the guano microbiota was previously unknown. In this study, we compared the postexposure microbiota community changes in bat guano collected immediately after defecation and stabilized at different time points, representing sample acquisition through active surveillance and capture techniques (0 hr postexposure) and indirect guano‐based sampling (1 and 12 hr postexposure), respectively. Our results suggest that bat guano is susceptible to significant microbiota shifts that are much more rapid (at least under arid conditions of the Colorado Plateau) than the days (Menke et al., 2015; Wong et al., 2016) or weeks (Dominianni et al., 2014; Flores et al., 2015; Menke et al., 2015) observed for fecal material of other mammals. This is surprising, but it does highlight the need for systematic species‐specific efforts to quantify fecal microbiota degradation before indirect surveillance methods can be properly applied on a large scale. From our work, moderate disruption in bacterial diversity can be expected even after 1 hr of exposure (largely due to anaerobic to aerobic environment shift). Importantly, we generally saw that the variation in relative composition of the guano microbiota within an individual at different time points was not as great as the variation between individuals, even after 12 hr. However, based on the current trajectory, we suspect that longer exposure may begin to disrupt this relationship. Thus, if the goal of a study is to assess the relative abundance of the core bat gut community or detect pathogens of interest via highly sensitive PCR‐based (both sequencing and TaqMan) methods, indirect sampling methods (e.g., tarp collection) appear to be very reliable, even at 12 hr of exposure. Alternatively, if the goal is to explore the gut microbiome composition (particularly the presence/absence of low‐frequency taxa), active or near‐immediate indirect (under 1 hr of exposure) methods would be preferable. It should be noted that our experiments were designed to test only the effects of exposure and therefore did not account for the higher potential for contamination that is likely to occur on a ground tarp. Further studies are needed to address this issue, but we expect that contamination will only be a major problem for low biomass samples; otherwise, the influence of any contaminants can be reduced by implementing minimum read thresholds when analyzing the data.

## CONFLICT OF INTEREST

None declared.

## AUTHOR CONTRIBUTION

FW and CLC collected fresh guano samples and were responsible for all bat capture via mist‐netting approach; DS performed field collection and sample stabilization; JC, DS, and CS performed DNA extraction and sequencing; TF, VYF, NP, and CH performed bioinformatics analyses; VYF and TF prepared the manuscript, with additional help from FW, CLC, DS, CH, JC, and CS. All authors contributed critically to the drafts and gave final approval for publication.

## DATA ACCESSIBILITY

Sequence read data analyzed in this study are deposited at the Sequence Read Archive (SRA, http://www.ncbi.nlm.nih.gov/sra) under SRA Study# SRP119282.
