# Supplementary material for: Guano exposed: Impact of aerobic conditions on bat fecal microbiota
Source: Ecol Evol. 2018 Apr 27;8(11):5563–74. doi: 10.1002/ece3.4084 (PMC6010783; doi:10.1002/ece3.4084)
Supplement: Supplementary file 1 [file ECE3-8-5563-s001.docx]

**APPENDIX:** QIIME Version 1.9.1 Protocol

**Fastq Demultiplexing**

**$ multiple_split_libraries_fastq.py -i INPUT_DIR -o OUTPUT_DIR \**

**--demultiplexing_method sampleid_by_file**

This script called the split_libraries_fastq.py script with default parameters:

--max_bad_run_length = 3

--min_per_read_length_fraction = 0.75

--sequence_max_n = 0

--phred_quality_threshold = 3

--max_barcode_errors = 1.5

**Open-reference OTU Picking**

OTUS were picked using the default UCLUST algorithm and the Greengenes reference set version 13_8 with 97% OTU representative sequence clusters

**$ pick_open_reference_otus.py -i INPUT_DIR -o OUTPUT_DIR \**

**--demultiplexing_method sampleid_by_file**

Default parameters were used:

--otu_picking_methods = uclust

--reference_fp = /Users/caporaso/.virtualenvs/qiime/lib/python2.7/site-packages/qiime_default_reference/gg_13_8_otus/rep_set/97_otus.fasta

--prefilter_percent_id = disabled

--minimum_failure_threshold = 100000

--percent_subsample = 0.001

--min_otu_size = 2

**Core Diversity Analysis**

**$ core_diversity_analyses.py -I \ otu_table_mc2_w_tax_no_pynast_failures.biom -o OUTPUT_DIR \ -m MAPPING_FILE -e 4800 -t rep_set.tre**
